# Potential Valorization of Edible Nuts By-Products: Exploring the Immune-Modulatory and Antioxidants Effects of Selected Nut Shells Extracts in Relation to Their Metabolic Profiles

**DOI:** 10.3390/antiox11030462

**Published:** 2022-02-25

**Authors:** Mohamed A. Salem, Nora M. Aborehab, Ahmed A. Al-Karmalawy, Alisdair R. Fernie, Saleh Alseekh, Shahira M. Ezzat

**Affiliations:** 1 Department of Pharmacognosy and Natural Products, Faculty of Pharmacy, Menoufia University, Gamal Abd El Nasr St., Shibin Elkom 32511, Egypt; 2 Department of Biochemistry, Faculty of Pharmacy, October University for Modern Sciences and Arts (MSA), Giza 12451, Egypt; naborehab@msa.edu.eg; 3 Department of Pharmaceutical Medicinal Chemistry, Faculty of Pharmacy, Horus University-Egypt, New Damietta 34518, Egypt; akarmalawy@horus.edu.eg; 4 Max Planck Institute of Molecular Plant Physiology, Am Mühlenberg 1, 14476 Potsdam-Golm, Germany; fernie@mpimp-golm.mpg.de; 5 Center for Plant Systems Biology and Biotechnology, 4000 Plovdiv, Bulgaria; 6 Pharmacognosy Department, Faculty of Pharmacy, Cairo University, Kasr El-Ainy Street, Cairo 11562, Egypt; shahira.ezzat@pharma.cu.edu.eg; 7 Department of Pharmacognosy, Faculty of Pharmacy, October University for Modern Sciences and Arts (MSA), Giza 12451, Egypt

**Keywords:** nuts, by-products, shells, metabolomics, immune-modulatory, molecular docking, SAR

## Abstract

The immune system is a potent army that defends our body against various infections and diseases through innate and adaptive immunity. Herbal medicine is one of the essential sources for enhancing immunity because of affordability, availability, minor side effects, and consumers’ preferences. Hazelnuts, walnuts, almonds, and peanuts are among the most widespread edible nuts that are rich in phenolics, fats, fibers, vitamins, proteins, and minerals. The potential of nut shells in phytoremediation has attracted increasing attention as a sustainable solution for waste recycling. Here, we determined the *in vitro* immune-modulatory activity as well as the metabolite profile of the four nut shell extracts. The addition of the extracts to LPS-stimulated macrophages, especially peanut and walnut shells, has downregulated the gene expression of AP-1, TNF-α, IL-8, iNOS, and COX-2 expression levels. Significant antioxidant capabilities and immune-modulatory effects have been traced for peanut shells. UPLC-MS metabolic profiling of the four nut shell extracts allowed the detection of a relatively high level of phenolic compounds in peanut shells. Intriguingly, a significant correlation between the antioxidant capacity and the total phenolic content was found, indicating the contribution of the phenolic compounds to the antioxidant properties and hence the immune-modulatory activity. Furthermore, molecular docking and structure–activity relationship (SAR) studies revealed kaempferol rutinoside and proanthocyanidin A5’ as potential iNOS inhibitors.

## 1. Introduction

Immunity is the ability of an organism to kill pathogens and fight infections through stimulation or raising the activity of any of its components [1]. There are two types of immunity; innate and acquired immunity. Innate (non-specific) immunity is the first natural immunological response present at the time of birth, occurring within minutes to hours, and has no immunological memory to pathogens, while acquired (adaptive) immunity is the second line of defense, it responds more slowly over a few days, and possesses immunological memory [2]. There are many factors such as age, malnutrition, certain medications such as corticosteroids, long-term stress, and excessive alcohol intake that reduce the immune response [3].

Abnormalities in either innate or adaptive immune responses can cause diseases. These diseases are usually caused by overactive immune responses (known as autoimmunity) such as type 1 diabetes mellitus or inadequate immune responses (called immunodeficiency) such as Acquired immunodeficiency syndrome (AIDS) [4]. Chronic diseases are considered a significant public health concern and constitute approximately 60% of all deaths in the world. Immunity is a key concept for epidemic control [5]. Till now, there is no proven treatment against pandemic viral infections so the only pathway available is to strengthen immunity defense through good nutrition and dietary intake [6]. 

Herbal medicine is widely used among people because of the low price, availability, cultural acceptability, and huge demand for organic products [7]. Nowadays, people use herbal medicine to boost their immunity [6]. Herbal medicine usage has to be based not only on ethnopharmacological indications but also on scientific evidences, including recent biologic screening methods in combination with analytical approaches such as metabolomics [7]. Recently, several drugs have been approved, which are of natural products or generated from natural ones, highlighting the significance of natural products in drug research and development [8]. 

Hazelnuts, walnuts, almonds, and peanuts are the most widespread edible nuts that are rich in fats, especially unsaturated fatty acid as oleic and linoleic acids, amino acids, sugars, starches, dietary fibers, minerals such as calcium, iron, and potassium as well as vitamins such as vitamin C, E and B [9,10]. Phenolic compounds are abundant in all four nuts by-products with various levels and derivatives [9]. Worldwide, hazelnut (*Corylus avellana* L., family Betulaceae) is considered to be one of the most well-known tree nuts. For both hazelnut kernels and other by-products such as skin, hard shell, and leaves, phenolics are considered the most abundant compounds in all hazelnut by-products, consequently highlighting hazelnut as a source of bioactive phytochemicals [11,12]. Their major biological activities that have been proved are antioxidant, anti-proliferative, and antimicrobial activities [11]. Walnut (*Juglans regia* Dode. family Juglandaceae) is rich in polyphenolic constituents, including phenolic acids, tannins, and flavonoids, demonstrating its pharmacological activities as antioxidants, antimicrobial, anti-inflammatory as well as its immunomodulatory activities [13]. Almond (*Prunus dulcis* (Mill.) D. A. Webb, family Rosaceae) is one of the most well-known nuts as a good source of nutrients, vitamins, and phytochemicals. The antioxidant activity of its high phenolic compounds is involved in its activity against cancer and aging [14]. Peanut or groundnut *(Arachis hypogaea,* family Fabaceae) is a species in the legume or “bean” family. Peanuts are known for high phenylpropanoid content, mainly stilbenes and flavonoids [15]. These compounds are involved in defense mechanisms against physical injuries and microbial contamination. For that, peanuts are considered a potent immunostimulant apart from their antimicrobial, antiviral, and antioxidant activities [16]. 

However, comparative studies on the chemical composition of the nut shells, their antioxidant activities and potential effects on immunomodulation are lacking. Therefore, the aim of the present study was to define the chemical composition and immune-modulatory activity of the ethanol extracts from hazelnut, walnut, almond, and peanut shells on THP-1 macrophages. The toxicity of the nut shell extracts on the differentiated THP-1 macrophage was also assessed using the MTT viability assay. Moreover, nut shell extracts were investigated for their *in vitro* antioxidant properties using 1,1-diphenyl-2-picrylhydrazyl radical (DPPH) and 2,2′-azinobis (3-ethylbenzothiazoline 6-sulfonate) (ABTS) as well as ferric reducing antioxidant potential (FRAP) assay. Furthermore, the molecular docking technique was used to investigate the recommended mechanism of action for the selected compounds accumulating in peanut shells as potential iNOS inhibitors. The resulting binding scores revealed kaempferol rutinoside, proanthocyanidin A5’, kaempferol glucoside, and proanthocyanidin A1 as potential candidates. 

## 2. Materials and Methods

### 2.1. Extraction

Hazelnut (*Corylus avellana*), walnut (*Juglans regia*), almond (*Prunus dulcis*), and peanut (*Arachis hypogaea*) were provided from a local market (Harraz for Food Industry and Natural Products, Egypt) in August 2020. All nuts were deshelled, and the shells were ground into powders using a commercial grinder. The powdered nut shells (500 g) were defatted and extracted by using *n*-hexane, and the defatted marc was extracted with ethanol (95%). Finally, the ethanolic extracts were evaporated under reduced pressure using the rotary evaporator at a temperature not exceeding 60 °C.

### 2.2. Total Phenolics Determination

Folin–Ciocalteu colorimetric method was used for the spectrophotometric determination of the total phenolic content in the tested extracts using gallic acid as a standard [17]. Samples solution of nut shell extracts were prepared at concentrations of 1 mg/mL in MeOH. Serial dilutions of gallic acid in methanol were prepared in concentrations 20 to 280 μg/mL. Distilled water (10 mL), Folin–Ciocalteu reagent (1.5 mL), and 20% Na_2_CO_3_ were added to 1 mL of each dilution of gallic acid and each sample. The volume in each case was completed to 25 mL with distilled water, followed by incubation for 30 min in dark at room temperature. The absorbance was measured at 630 nm for 6 replicates at each concentration and was expressed as mg gallic acid equivalent (GAE) [17].

### 2.3. Total Flavonoids Determination

AlCl_3_ colorimetric method was applied for the spectrophotometric determination of the total flavonoids content in the tested extracts using rutin as a standard [18]. Standards for total flavonoids were prepared by preparing rutin solution of 1 mg/mL in methanol. Samples solution of nut shell extracts were prepared at concentrations of 2.5 mg/mL in EtOH. Certain amount of the extract (125 µL) was added to 75 μL of a 5% sodiumnitrite solution then, and left for 6 min, 150 μL of AlCl_3_ (10%) was added then left for 5 min, after that 750 µL of NaOH (1 M) was mixed with them. The volume was adjusted with distilled water to 2.5 mL. The mixture turned pink after incubation for 15 min, and the absorbance was measured at 420 nm for 6 replicates at each concentration. The total flavonoids content was expressed as mg rutin equivalent [18]. 

### 2.4. Determination of Total Antioxidant Activity Using DPPH ASSAY

Samples were dissolved in EtOH to prepare 1000 and 100 µg/mL solutions to determine the range of their IC_50_ [19]. Samples that showed IC_50_ in the initial step were used to prepare 5 serial dilutions. Trolox serial dilutions were prepared in MeOH as follows 50, 40, 30, 20, 15, 10, and 5 µM. The method of Boly et al. 2016 was then applied, where 100 µL of DPPH reagent (freshly prepared as 0.1% in methanol) was added to 100 µL of each sample in 96 wells plate (*n* = 6), the solutions were kept in the dark at room temperature. After 30 min. the resulting colors were measured at 540 nm. Data are represented as means ± SD according to the following equation:

$$\mathrm{percentage}\;\mathrm{inhibition}=(\frac{\mathrm{Average}\;\mathrm{absorbance}\;\mathrm{of}\;\mathrm{blank}-\mathrm{average}\;\mathrm{absorbance}\;\mathrm{of}\;\mathrm{the}\;\mathrm{test}}{\mathrm{Average}\;\mathrm{absorbance}\;\mathrm{of}\;\mathrm{blank}})\times 100$$

Microplate reader FluoStar Omega was used, Microsoft Excel^®^ was used for data analysis, and Graph pad Prism 5^®^ to calculate IC_50_.

### 2.5. Determination of Total Antioxidant Activity Using FRAP Assay

Ten serial dilutions of Trolox were prepared in the concentrations of 4000, 3000, 2000, 1000, 800, 600, 400, 200, 100, and 50 µM for standards. All samples were prepared in concentrations of 1 mg/mL. The method of Benzie and Strain was used with minor modifications [20]. A freshly prepared solution of 2,3,5-Triphenyltetrazolium chloride (TPTZ) reagent (190 µL) was added to 10 µL of the sample in 96 wells plate (*n* = 6), the reaction was incubated in the dark at room temperature. After 30 min, the resulting blue color was measured at 593 nm. Data were represented as means ± SD [20,21].

### 2.6. Determination of Total Antioxidant Activity Using ABTS Assay

The ABTS assay was carried out according to the method of Arnao et al., with minor modifications [21]. Samples were used in a concentration of 0.2 mg/mL in ethanol. Trolox was used as a standard in serial dilutions (50–600 µM). ABTS was prepared by adding 192 mg to distilled water in a 50 mL volumetric flask. One mL of ABTS solution was mixed with 17 µL of 140 mM potassium persulphate then left in the dark for 24 h. Hereafter, the prepared solution (1 mL) was completed with methanol to 50 mL, then 190 µL of the freshly prepared ABTS reagent were mixed with 10 µL of the sample/compound in 96 wells plate (*n* = 4), and kept in the dark for 120 min. The decrease in ABTS color intensity was measured at 734 nm. Data is represented as means ± SD according to the following equation:

$$\mathrm{percentage}\;\mathrm{inhibition}=(\frac{\mathrm{Average}\;\mathrm{absorbance}\;\mathrm{of}\;\mathrm{blank}-\mathrm{average}\;\mathrm{absorbance}\;\mathrm{of}\;\mathrm{the}\;\mathrm{test}}{\mathrm{Average}\;\mathrm{absorbance}\;\mathrm{of}\;\mathrm{blank}})\times 100$$

### 2.7. Ultra-Performance Liquid Chromatography Coupled with Tandem Mass Spectrometry (UPLC-MS/MS) Analysis

The dried extracts (10 mg) were re-suspended in 2 mL of 50% methanol/water. The samples were vortexed before they were subjected to centrifugation for 1 min at 5000× *g*. 100 µL of the supernatant was transferred to LC glass vials. An untargeted platform consisting of reversed-phase (RP-C18) ultra-performance liquid chromatography (UPLC) coupled to high-resolution tandem mass spectrometry (MS/MS) analysis. Mass spectrometry analysis was performed using positive (+ESI) and negative (−ESI) ion modes [22].

### 2.8. Biological Screening

The human monocyte cell line (THP-1, ATCC TIB-202) was cultured using Roswell Park Memorial Institute media (RPMI-1640, Lonza, Belgium) supplemented with 10% fetal bovine serum (Gibco, Thermo Fisher Scientific Inc., Bremen, Germany), and 1% penicillin–streptomycin (Lonza, Belgium). Phorbol 12-myristate 13-acetate (PMA) was used for induction of Monocyte-Macrophage differentiation in the THP-1 monocytic cells. The cells were treated with 100 ng/mL PMA for 48 h, and the differentiated macrophages were then cultured in fresh RPMI-1640 media for 24 h before the experimental use.

#### 2.8.1. *In Vitro* Cytotoxicity Using MTT Assay

The MTT method was used to assay *in vitro* cytotoxicity of the extracts. First cultures were transferred from incubator into sterile area, and then MTT vial was reconstituted to be used with 3 mL of medium. After that, the cultures were returned to incubator for 2–4 h. Prior to the incubation, MTT [M-8910] was added in equal amounts to volume of original culture medium, followed by mild mixing using gyratory shaker to enhance the dissolution. The absorbance was measured at wavelength 570 nm using spectrophotometer. Mean inhibitory concentration (IC_50_) of all the extracts was calculated as the mean of 3 independent experiments

#### 2.8.2. LPS Induction in THP-1 Macrophage

After 48 h of THP-1 macrophage differentiation using PMA, the media was aspirated, and the cells were incubated for a further 24 h in PMA-free medium. On day 4, the cells were incubated with IC_50_ of the tested extract with LPS (Sigma-Aldrich) (0.5 μg/mL) for an additional 24 h. The control was performed using media supplemented with 0.5 μg/mL LPS only

#### 2.8.3. RNA Extraction

After the previous incubation, the cells were collected for total RNA extraction using the RNeasy mini kit (Qiagen, GmbH, Hilden, Germany) according to the manufacturer’s instructions.

#### 2.8.4. Quantitative Polymerase Chain Reaction (q-PCR)

Primers for these genes; “NF-Kβ” gene, “COX-2” gene, “iNOS” gene, “SP-1” gene, “AP-1” gene, and the housekeeping gene “β-actin” (Thermo Fisher Scientific Inc., Germany) were produced (Table S1). cDNA synthesis and PCR amplification were performed using the iScript TM One-Step RT-PCR Kit with SYBR^®^ Green (Bio-Rad Laboratories, Hercules, CA, USA) according to manufacturer instructions on Rotor-Gene Q real-time PCR cycler. The relative expression of tested mRNA genes was calculated using real-time data. In order to facilitate the normalization of all results, the β-actin gene was used. Finally, using the following equation, values were presented as fold changes: 2^ΔΔCT [23].

#### 2.8.5. Cytokine Determination by ELISA

Using the same induction method described previously, the concentration of the released cytokines (TNF-α, IL-1β, IL-6, IL-8, and IL-10) were measured in the supernatant using the respective ELISA kit according to the manufacturer’s instructions using ROBONIK P2000 Elisa Reader.

### 2.9. Docking Studies

The selected compounds accumulating in peanut shells were subjected to molecular docking using the MOE 2019.012 suite [24,25] to recommend them as potential iNOS inhibitors. Furthermore, the co-crystallized native inhibitor (chlorzoxazone, CLW) was used as a reference standard.

#### 2.9.1. Preparation of the Selected Compounds from the Studied Four Nut Shell Extracts

The selected compounds accumulating in peanut shells were downloaded from the database of PubChem website (https://pubchem.ncbi.nlm.nih.gov/) (accessed on 10 December 2021). Then, each compound was copied and pasted to the MOE program to be prepared for the docking process following the previously discussed steps [26,27]. Finally, all the prepared compounds, including the co-crystallized iNOS-inhibitor (CLW), were inserted in a single database and saved as (.mdb file) to be uploaded during the docking step.

#### 2.9.2. iNOS Receptor Pocket Preparation

The X-ray structure of the iNOS receptor (ID:1M8D) [28] was downloaded from the PDB website, inserted into the MOE program, and subjected to the preparation steps described before [29,30].

#### 2.9.3. Docking of the Mentioned Database into the iNOS Binding Pocket

The previously prepared database was inserted into the docking process, applying the general methodology described in detail earlier [31,32]. Notably, the scoring value, RMSD, and binding mode were the most important factors used for the selection of the best pose for each studied compound [33,34]. Furthermore, it is worth mentioning that a program validation process was performed by applying a separate re-docking process for the co-crystallized inhibitor of iNOS. The obtained RMSD values (<2) confirmed the validity of the docking program [35,36]. 

### 2.10. Data Analysis and Visualization

LC/MS data analysis was performed by using the ToxID 2.1.2 and Xcalibur 2.1 software package (Thermo Fisher Scientific Inc., USA). All the obtained data were analyzed and correlated using Metaboanalyst (https://www.metaboanalyst.ca/ (accessed on 15 June 2020)) [37]. Statistical analysis was performed using the GraphPad Prism software (version 6; GraphPad Software, Inc., San Diego, CA, USA). The data are presented as mean ± standard deviation. Statistical comparison between groups was made using One-Way-ANOVA followed by Tukey posttest for multiple comparisons. Differences were considered significant when *p* < 0.05. The proposed molecular mechanism scheme was created with the help of BioRender.com.

## 3. Results

### 3.1. Total Phenolic and Flavonoid Contents

**Figure 1:**
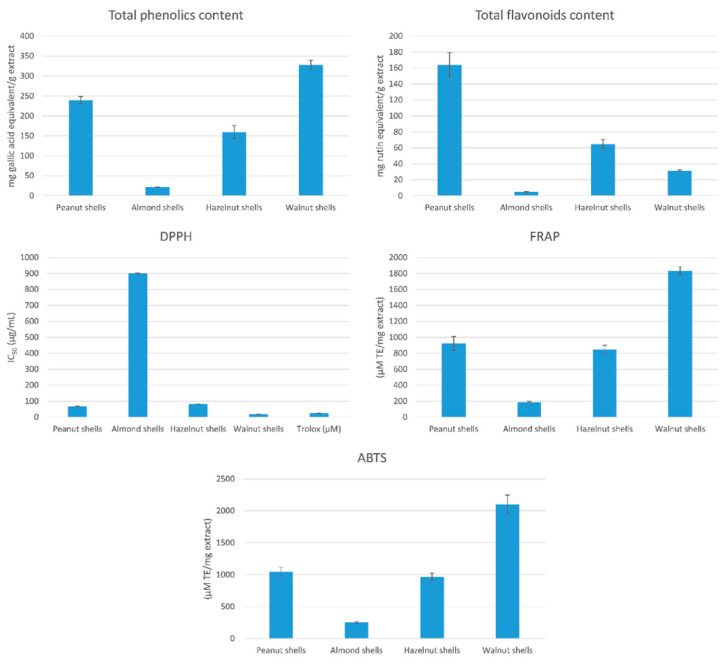
Evaluation of the total phenolics contents and antioxidant potential of nut shell extracts.

In order to investigate the chemical profile and biological activities of hazelnut, walnut, almond, and peanut shells, ethanolic extracts from powdered and defatted shells were prepared. Using the Folin–Ciocalteu method, walnut shell (328.09 mg gallic acid equivalent/g extract) and peanut shells (240.08 mg gallic acid equivalent/g extract) had the highest amount of total phenolics compared to hazelnut (159.70 mg gallic acid equivalent/g extract) and almond (21.46 mg gallic acid equivalent/g extract) shells (Figure 1). Peanut (164.03 mg rutin equivalent/g extract) and hazelnut (64.83 mg rutin equivalent/g extract) had the highest number of total flavonoids compared to walnut (31.00 mg rutin equivalent/g extract) and almond (4.75 mg rutin equivalent/g extract) shells (Figure 1).

### 3.2. Determination of Total Antioxidant Activity

Results of antioxidant activity using DPPH free radical scavenging method indicated that the IC_50_ of walnut, peanut, hazelnut, and almond shells were 18.33, 67.72, 82.11, and 900.9, respectively (Figure 1). This result highlights the high DPPH scavenging activity of walnut and peanut shell extracts. Using FRAP Assay, results showed that walnut (1833.96 µM TE/mg extract) and peanut (924.32 µM TE/mg extract) shells had higher activity compared to hazelnut (848.07 µM TE/mg extract) and almond (190.37 µM TE/mg extract) shells. Using ABTS Assay, walnut (2101.44 µM TE/mg extract), peanut (1048.38 µM TE/mg extract), hazelnut (966.08 µM TE/mg extract), and almond (256.44 µM TE/mg extract) had the same potency as in FRAP assay.

A regression analysis was performed, as evaluated by correlation coefficient (R), in order to correlate the results of the total antioxidant activities in the used methods with the total phenolic contents. Significant correlations were found between the various methods used to determine the antioxidant potential, especially between FRAP and ABTS (R = 0.998) (Figure S1). Results of the antioxidant capacities were also correlated to the total phenolic contents. A strong correlation was found between the antioxidant potential, as determined by the ABTS and FRAP assays, and total phenolic contents (R = 0.962 and R = 0.957, respectively).

### 3.3. Evaluation of the Cytotoxicity Using MTT Assay and Gene Expression by q-PCR

**Figure 2:**
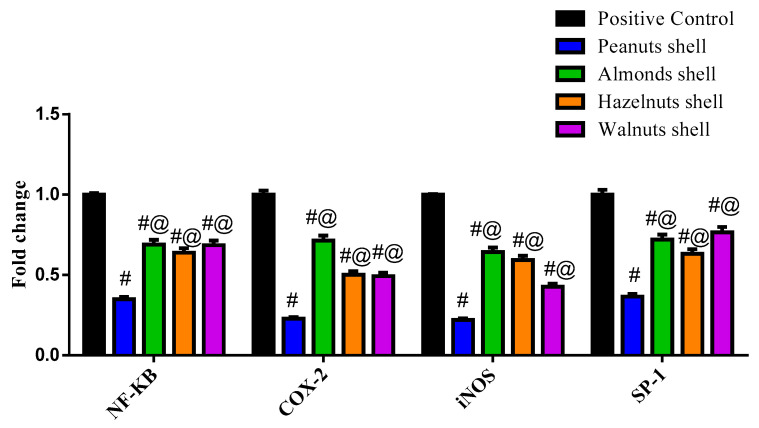
The biochemical effect of different nut shell extracts on NF-KB, Cox-2, iNOS and SP-1 expression levels. ^#^ Significant from Positive control at *p* < 0.0001, ^@^ Significant from Peanut shells at *p* < 0.0001.

**Figure 3:**
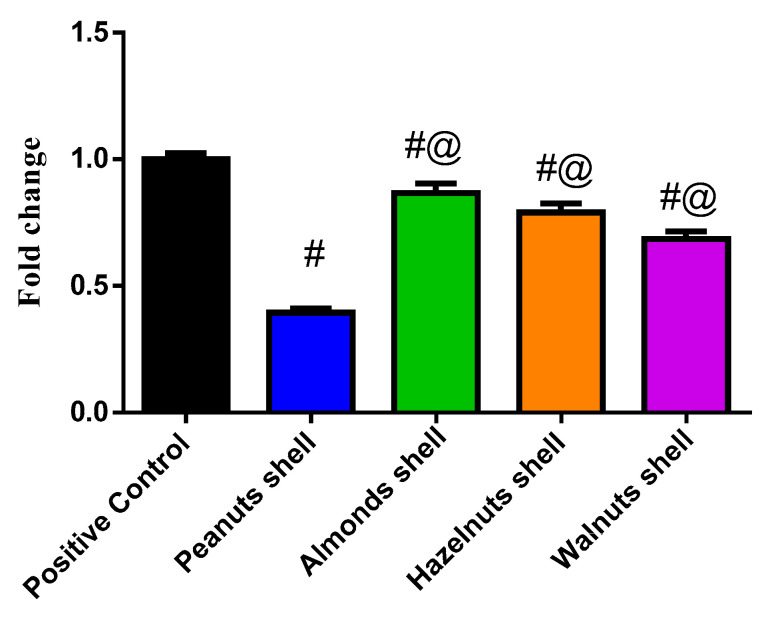
The biochemical effect of different nut shell extracts on AP-1 expression level. ^#^ Significant from Positive control at *p* < 0.0001, ^@^ Significant from Peanut shells at *p* < 0.0001.

The effect of each nut shell extract on THP-1 cell viability was investigated. The results showed that all the tested samples did not exhibit cytotoxicity (Figure S2). The mRNA expression of the inflammation-related enzymes (iNOS and COX-2) and the transcription factors (NF-κB, AP-1, and SP-1) were upregulated in THP-1 macrophages stimulated by LPS (Figure 2 and Figure 3). Co-treatment of the peanuts, almonds, hazelnuts, and walnut shell extracts attenuated the expression level of COX-2, iNOS, NF-κB, AP-1, and SP-1 compared to the positive control group at *p* < 0.0001. Peanut shell extracts significantly decreased the expression of iNOS to 0.221 ± 0.01, COX-2 to 0.228 ± 0.010, NF-κB to 0.35 ± 0.015, SP-1 to 0.366 ± 0.016 and AP-1 to 0.394 ± 0.017 when compared to other nut shell extracts at *p* < 0.0001.

### 3.4. Cytokines Determination by ELISA

Treatment of THP-1 cells by each nut shell extract resulted in a significant reduction of all cytokines compared to the positive control group at *p* < 0.0001. Peanut shell extracts showed a prominent effect in reducing the levels of IL-1β (64.4 ± 4.6 pg/mL), IL-6 (113.2 ± 5.46 pg/mL), IL-8 (135.6 ± 13.2 pg/mL) and TNF-α (83.49 ± 4.35 pg/mL) compared to other nut shell extracts at *p* < 0.0001 (Table 1).

### 3.5. Metabolite Identification and Multivariate Data Analysis

An ultra-high performance liquid chromatography coupled to high-resolution mass spectrometry (UHPLC/HRMS) analysis was used for the untargeted analysis of metabolites from nut shell extracts. Identification of metabolites was based on comparing the MS and MS/MS data to an in-house database, previously published data on nuts, as well as common MS databases such as Human Metabolome Database (http://www.hmdb.ca/ (accessed on 7 January 2019)), MassBank (www.massbank.jp (accessed on 20 January 2019)) and METLIN (http://metlin.scripps.edu (accessed on 25 March 2019)). An accuracy error of 10 ppm was set in the MS search, and the fragments were verified in MS/MS search. 

**Figure 4:**
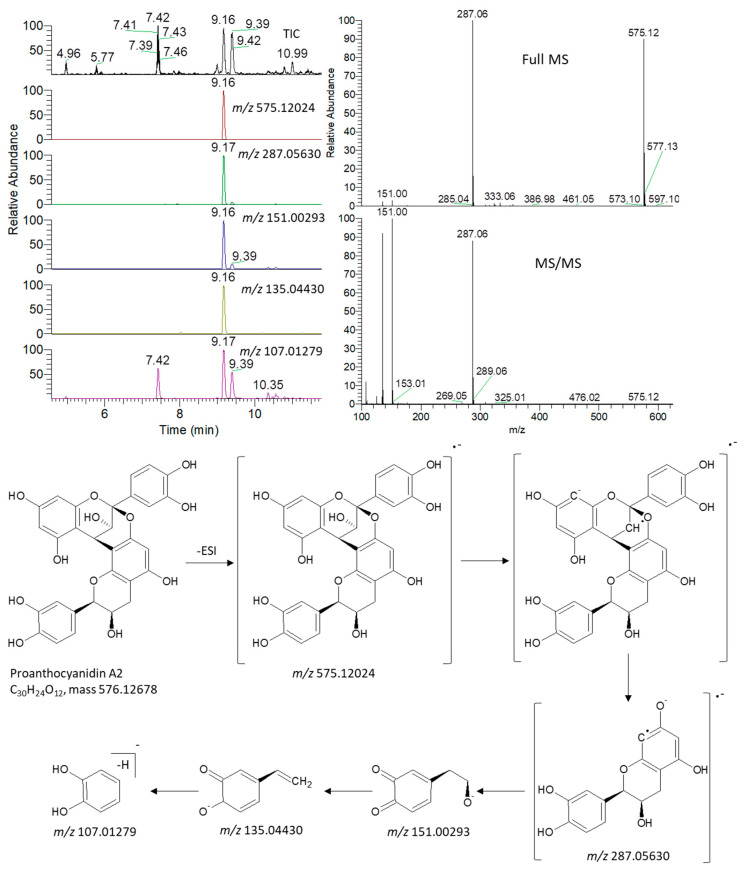
Total ion chromatogram (TIC) and extracted ion chromatograms (EIC) of the peak at 575.12024 *m*/*z* representing proanthocyanidin A2 measured by UPLC/MS in negative ionization mode with schematic diagram showing the production of fragment ions during MS/MS analysis.

In order to illustrate the identification process, an exemplary detail is provided for the identification of proanthocyanidin A2 (Figure 4). The extracted ion chromatograms (EIC) of its deprotonated adduct were detected at *m*/*z* 575.12024 [M − H]^−^ and a retention time of 9.16 min. The predicted molecular formula for this adduct was C_30_H_23_O_12_. Searching the molecular formula against common MS databases showed that the formula could be tentatively assigned to proanthocyanidin A2. Characteristic fragment ions were detected at the same retention time as their precursor ion. Quinone methide (QM) reaction resulted in fragmentation between two epicatechin subunits forming different QM ions (*m*/*z* 287.05630), with its dissociation yielding characteristic ions at *m*/*z* 151.00293, 135.04430, and 107.01279 [38,39] (Figure 4). Our analysis revealed the presence of 93 identified metabolites, including flavonoids, anthocyanins, amino, phenolic, and organic acids (Table S2).

**Figure 5:**
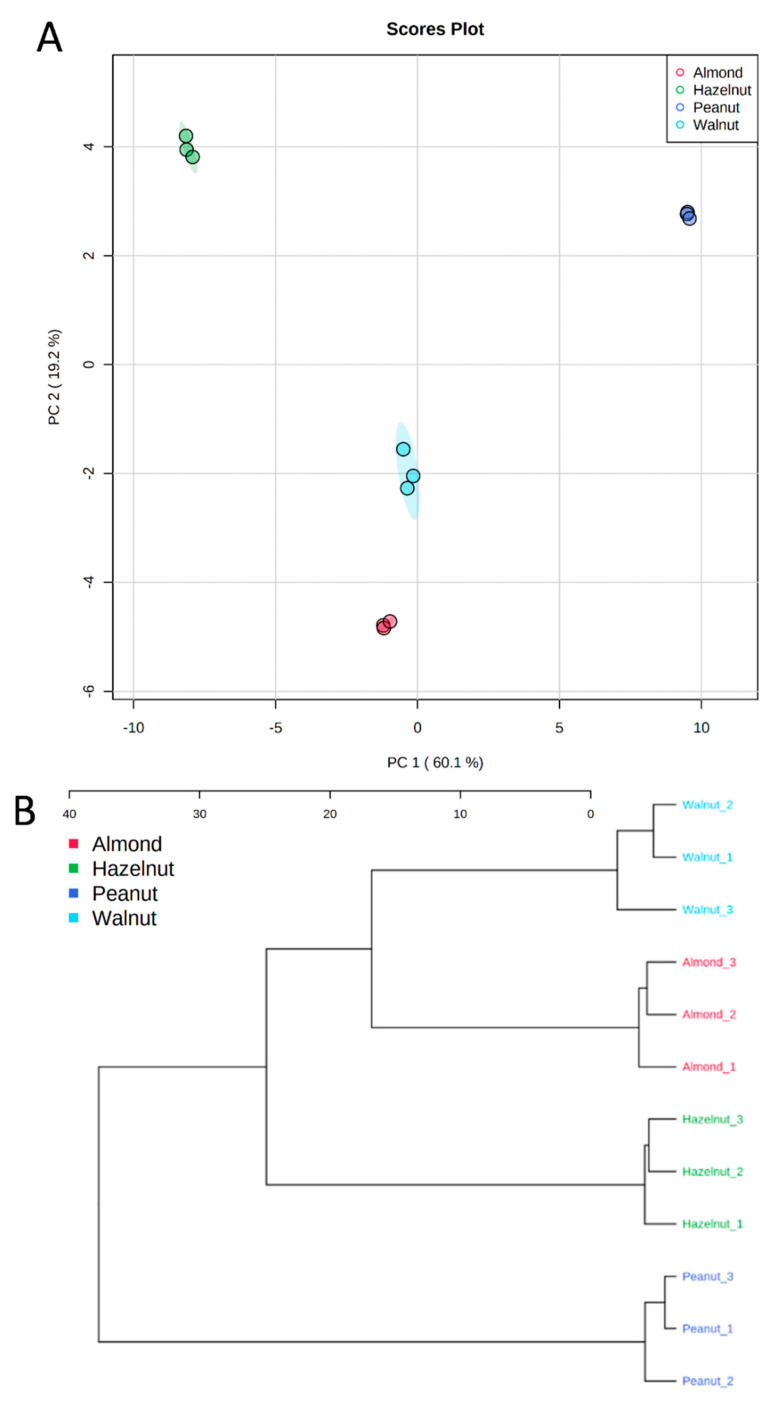
Principal component analysis (PCA) scores plot (**A**) and hierarchical cluster analysis (HCA) (**B**) of nut shell extracts based on the UPLC/MS data.

**Figure 6:**
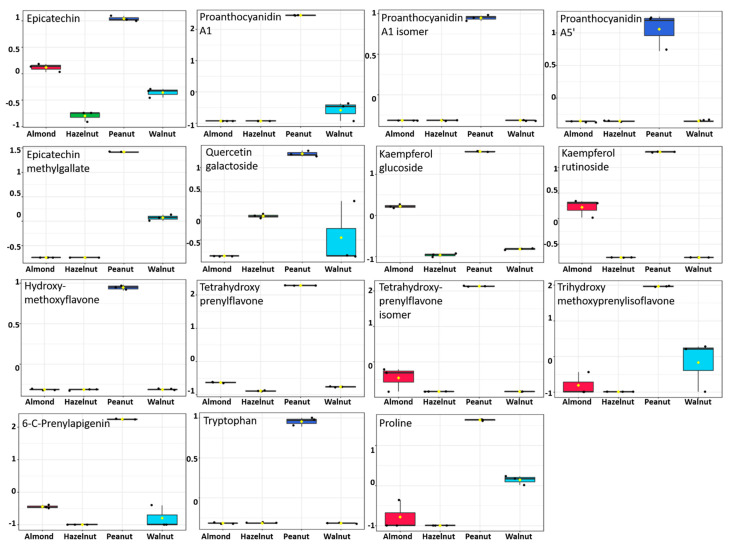
Metabolites with significant accumulation in peanut shell extract. The *y*-axis represents the scaled and log_10_-transformed values of metabolite abundance.

In order to verify the metabolomic differences between the tested nutshell extracts, multivariate data analysis was performed. The principal component analysis (PCA) showed a clear separation of peanut shell extracts, with about 80% of the variance explained by the first two PCs (Figure 5). Hierarchical cluster analysis (HCA) revealed two main clusters. The first cluster consisted of peanut shell extract that was discriminated from other shell extracts (Figure 5). The second cluster consisted of hazelnut shell extract that was discriminated from other shell extracts. Several compounds were identified and contributed to the clustering of peanut shells from others, such as proanthocyanidins (proanthocyanidin a1, proanthocyanidin a5’, epicatechin, epicatechin methylgallate), flavonoids (kaempferol, quercetin, and apigenin derivatives), and amino acids (tryptophan and proline) (Figure 6).

### 3.6. Docking Studies

A molecular docking study was carried out for the selected compounds accumulating in peanut shells against iNOS due to their main role in inflammation and immunomodulation pathways. Observing the iNOS X-ray structure revealed the presence of its co-crystallized inhibitor (chlorzoxazone, CLW) as one of its crucial inhibitors. It showed the formation of two H-bonds with the most important amino acids (Met368 and Trp366) of the receptor pocket. Moreover, it was stated that Glu371 represents a third important amino acid for the inhibitory effect of the iNOS receptor by introducing the required conformational change as well. Based on the above, we can conclude that the recommended inhibitor for iNOS is preferred to bind Met368, Trp366, and/or Glu371 amino acids [28]. The obtained binding scores of the studied candidates and their binding modes with the iNOS pocket amino acids were represented in Table 2 and Table 3. Furthermore, their 2D binding interactions were provided in Table S3. Herein, the molecular docking of the selected compounds accumulating in peanut shells revealed the following descending order: kaempferol rutinoside > proanthocyanidin A5’ > kaempferol glucoside > proanthocyanidin A1 > epicatechin methyl gallate > quercetin galactoside > trihydroxy methoxy-prenyl isoflavone > tetrahydroxy prenylflavone > 6-C-prenylapigenin > hydroxy-methoxy flavone > epicatechin > tryptophan > co-crystallized inhibitor (CLW) > proline.

### 3.7. Structure–Activity Relationship (SAR)

**Figure 7:**
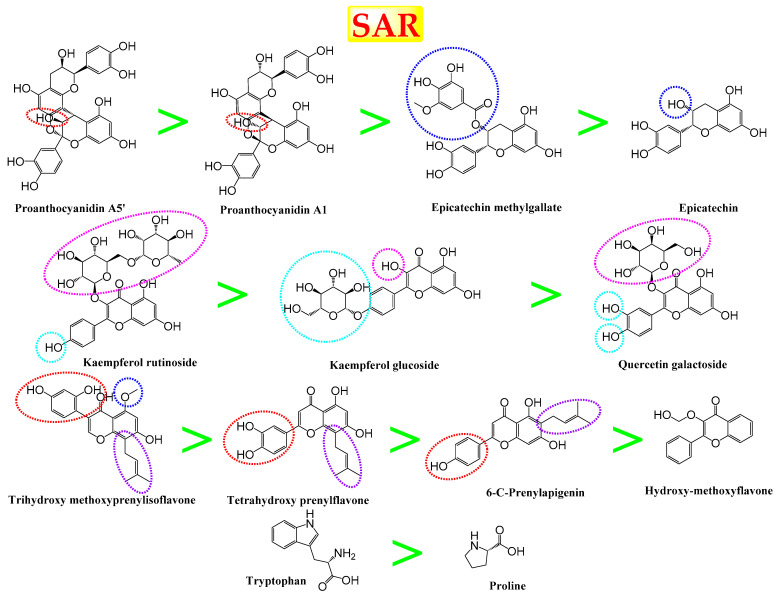
Structure–Activity relationship (SAR) study of the selected compounds of the studied four nut shell extracts as potential iNOS inhibitors.

Observing the binding score results of the selected compounds as potential iNOS inhibitors in relation to their chemical structures revealed interesting findings (Figure 7). The introduction of the β-OH group in proanthocyanidin A5’ achieved a superior iNOS inhibitory activity compared to its α-position in proanthocyanidin A1. On the other hand, the presence of the methyl gallate group in position seven (epicatechin methyl gallate) showed a better iNOS inhibition compared to the presence of the OH group in the same position (epicatechin). Additionally, the introduction of both rhamnose and glucose units in position three of kaempferol rutinoside inhibited the iNOS receptor more efficiently than the presence of only glucose unit in position four of kaempferol glucoside. However, the presence of a galactose moiety in position three showed a lower iNOS inhibition ability compared to that of kaempferol rutinoside. Furthermore, the presence of the 2-methylbut-2-ene moiety in position eight of both trihydroxy methoxy prenyl isoflavone and tetrahydroxy prenylflavone attained a better iNOS inhibition than its presence in position six (6-C-prenylapigenin). Furthermore, the presence of 2,4-dihydroxy phenyl moiety in position three of trihydroxy methoxy prenyl isoflavone showed a superior binding affinity compared to the 3,4-dihydroxy phenyl one in position two of tetrahydroxy prenylflavone followed by the p-hydroxyphenyl moiety in position two of 6-C-prenylapigenin. Finally, tryptophan amino acid moiety was found to be superior in iNOS inhibition compared to proline amino acid. 

## 4. Discussion

Environmental pollution due to different agro-industrial wastes is a worldwide concern. Environmental contamination is a significant risk that has a negative effect on public health. Consequently, recent research is focusing on the effective use of agro-industrial wastes in order to eliminate their harmful effects on living organisms and the use of such wastes for the production of biologically active pharmaceutical products [40]. For this reason, we aimed in our article to use the shells, which are the waste products of commonly used nuts, for the production of biologically active products.

To our knowledge, this is the first demonstration to report the immune-modulatory activities of peanut, almond, hazelnut, and walnut shell extracts using an *in vitro* model. This effect is probably mediated through the down expression of iNOS and COX-2 and the transcription factors NF-κB, AP-1, and SP-1. The chemical characteristics of the antigen stimulate the immunological response. Bacteria are well-known monocyte and macrophage inducers that play a key role in the onset of several host responses [41]. Their activity contributes to systemic inflammatory events, such as cytokine gene upregulation, which leads to cytokine protein release into the blood. IL-8 and TNF-α are pro-inflammatory cytokines that play a key role in the initiation and progression of inflammation. Interleukin 8 (IL-8) is a strong anti-inflammatory cytokine that plays a key role in mediating the host anti-inflammatory response and, as a result, in preventing host damage and maintaining normal tissue homeostasis [41].

After stimulation, the inflammation-related cytokine TNF-α is released in high levels, and its production is linked to the release of other cytokines, including IL-8 and inflammation-related enzymes (iNOS and COX-2). Certain transcription factors have been linked to the receptor-mediated production of cytokine and enzyme genes, either directly or indirectly. The transcriptional factor of cytokines and enzyme genes is regulated by the transcription factor (NF-κB). Assays that identify changes in cytokines, enzymes (iNOS and COX-2), and NF-κB are crucial for exploring and evaluating the inflammation-related immunological responses of natural products. THP-1 monocytes/macrophages are a sensitive, distinctive, and precise in vitro cell model for studying inflammation-related immune responses to various bioactive products [41].

Nuts are consumed as dietary supplements, having disease-preventing capabilities due to their immunomodulating properties [14,42]. A previous study has found that using a crude polysaccharide extract from *Prunus dulcis* seed cell wall material stimulates murine lymphocytes. This was evaluated by the expression of lymphocyte activation markers *in vitro* and *in vivo*, as well as the proliferation of spleen mononuclear cells in culture. B-cells were more stimulated than T-cells by the lymphocyte stimulatory impact. There was no evidence of cytotoxicity caused by the polysaccharide fractions [43]. Another study showed that hazelnut-hydrolyzed peptides of *Coryllus hytrophylla* fish have significant in vivo cell-mediated and humoral immunity immunomodulatory effects [44]. On the other hand, the ingestion of *Coryllus avellana* leaves by sheep did not improve the *in vitro* activation and proliferation index of PBMC, which in turn have shown no effect on the cellular immune response of sheep [45]. Furthermore, it was shown that feeding *Arachis hypogea* extracts to rohu fish resulted in a significantly higher survival percentage when compared with the control, suggesting that the extracts of *Arachis hypogea* have immunostimulant activity by stimulating both specific and non-specific immunity at higher concentrations [46]. Additionally, walnut oligopeptides could significantly improve both innate and adaptive immunity through activating natural killer cells and improving *in vivo* production of immunoglobulin, indicating that WOPs could be great immune boosters at a dose range of 110–440 mg/kg [47]. The aforementioned studies focused on the evaluation of nuts edible parts, while the comparative chemical as biological evaluation of nut shells was not deeply investigated.

The results presented here showed that walnut and peanut shells had the highest amount of total phenolic and flavonoids contents. Since the cell inflammation is accompanied by an elevation in the oxidative stress response and reductions of its intracellular pH, the determination of the antioxidant capability of each shell extract was essential. Previous studies have demonstrated the potential antioxidant activity of hazelnuts [48], walnuts [13], almonds [49], and peanuts [42,50]. In the present study, the antioxidant activity of shells was measured by various chemical assays: DPPH, FRAP, and ABTS, highlighting their significant antioxidant activity, especially peanut shells, which is correlated to their high phenolic content. The trend for the antioxidant activities of the tested nut shell extracts did not vary between DPPH, FRAP, and ABTS scavenging capabilities giving comparable results. The highest correlations for the scavenging capabilities were found between FRAP and ABTS assays, consistent with previous studies [51,52]. Significant correlations were also found between the antioxidant activities and total phenolic contents, indicating the significant contribution of phenolic compounds to their antioxidant potential, consistent with previous studies [52,53,54]. 

NO is a free radical generated by nitric oxide synthases from L-arginine. NO is a cellular mediator that has a role in both physiologically and pathologically processes. It can generate or modify intracellular signals in sufficient quantities, impacting the activity of immune cells, tumor cells, and resident cells in various tissues and organs. Meanwhile, its uncontrolled release can induce inflammatory damage to the target tissue. The increased amount of NO produced by iNOS contributes to the inflammatory process and interacts with other inflammatory mediators synergistically; therefore, inhibition of iNOS activity or downregulation of iNOS expression may help to reduce the inflammation [55]. The effects of shell extracts on mRNA expression of transcription factors (NF-κB, SP-1, and AP-1) and inflammation-related enzymes (iNOS and COX-2) were tested in THP-1 macrophages stimulated by LPS. Co-treatment of peanut shells decreased the mRNA expression level of NF-κB, which led to downregulation of Cox-2 and iNOS and significant reduction of the IL-1β, IL-6, IL-8, and TNF-α. Moreover, it downregulated the production of AP-1, which led to a significant reduction of the IL-8 and TNF-α. A previous study showed that peanut shell extract (PSE) significantly alleviated inflammatory bowel disease (IBD) symptoms and reduced the inflammation in a rodent model of colitis as it markedly reduced the levels of pro-inflammatory cytokines as TNF-α and IL-6 [56]. Peanut and walnut shells have shown significant results throughout this study, revealing their significant antioxidant activity and their promising immunomodulatory activity. Specifically, peanut shells showed great importance as a source of bioactive compounds in comparison to other included nut shells.

The inflammatory response begins with the production of pro-inflammatory cytokines, including TNF-α and IL-1β, which both stimulate tissue repair and regeneration. Several signaling pathways, including NF-Kβ and Mitogen-activated protein kinase (MAPK), are all involved in the generation of inflammatory modulators [57]. The phosphorylation of p38 MAPK was decreased by the treatment of ergosterol peroxide, which in turn decreases the mRNA expression of cytokines (IL-1β, IL-6, IL-12, TNF-α, IFN-α, IFN-β, Mx1, and PKR); therefore, the treatment regulates the host immune responses by downregulating NF-κB and p38/MAPK signaling pathways activation in vitro [58]. Interleukin 10 (IL-10) is an anti-inflammatory cytokine that plays a key role in limiting the immunological response of the host to infections, minimizing harm to the host, and maintaining normal tissue homeostasis [59]. Peanut shells were able to increase IL-10 levels, which decrease TNF-α, IL-1β, and IL-8 levels in LPS induced THP-1 cells; these results come in agreement with a study carried out by Chanput et al., 2010 where the co-stimulation of LPS with either quercetin, citrus pectin, or barley glucan in THP-1 monocytes and macrophages showed a different immunomodulatory activity on gene expression of IL-6, IL-10, IL-8, TNF- α, IL-1β and inflammation-related enzymes (iNOS and COX-2) [60]

Phenolic compounds and flavonoids were presented with significantly high amounts in peanut shells. Moreover, peanut shells have demonstrated significant antioxidant activity throughout all accompanied assays, in addition to its potential in the downregulation of both TNF-α and IL-8, apart from its effect in attenuating expression levels of inflammation accompanied enzymes iNOS and COX-2 as well as transcription factors AP-1, SP-1, and NF-κB. The metabolic profile demonstrated that epicatechins and flavonoids might contribute to these results, which in turn can be considered as very promising immunomodulatory compounds.

There is no doubt that computational chemistry is one of the most crucial methods for drug discovery and development nowadays. It helps to save more time and effort in the long way of new drug approval [61,62]. Molecular docking is considered to be the most applied computational technique to describe and evaluate the binding interactions and modes of the tested molecules within a specific receptor [63,64,65]. Analyzing the docking revealed that the co-crystallized CLW inhibitor showed a greatly similar binding mode to that of its native co-crystallized one. It formed two H-bonds with Trp366 and Met368 amino acids at 2.97 and 3.04 Å, respectively, and achieved a binding score of −4.78 kcal/mol. However, kaempferol rutinoside attained a binding score of −9.96 kcal/mol with the formation of one H-bond with Trp366 at 2.82 Å and two H-bonds with Met349 at 3.36 and 3.57 Å. On the other hand, proanthocyanidin A5’ showed a binding score of -8.67 kcal/mol and formed two H-bonds with Trp366 and Glu371 amino acids at 2.85 and 3.02, respectively. Moreover, the kaempferol glucoside binding score was found to be −7.82 kcal/mol and formed three H-bonds with Trp366 amino acid at 3.10, 3.13, and 3.24 Å. In addition, the proanthocyanidin A1 docking score was −7.73 kcal/mol and formed only one H-bond with Met368 amino acid at 3.01 Å. Furthermore, epicatechin methyl gallate showed the formation of one H-pi bond with Trp366 and one H-bond with Met368 amino acids at 3.76 and 4.25 Å, respectively. Its binding score was −7.54 kcal/mol. Moreover, quercetin galactoside showed a binding score of −7.47 kcal/mol with the formation of only one H-bond with Trp366 amino acid at 2.73 Å. Obviously, based on the binding scores and modes, we can conclude that all of the tested compounds -except for proline amino acid- showed superior results compared to that of the docked co-crystallized inhibitor (CLW). This represents highly recommended binding affinities of the tested selected compounds from the peanut shells towards the binding pocket of the iNOS receptor. Furthermore, this may recommend greatly predicted intrinsic activities for the tested compounds as well, especially for kaempferol rutinoside, proanthocyanidin A5’, kaempferol glucoside, and proanthocyanidin A1. 

**Figure 8:**
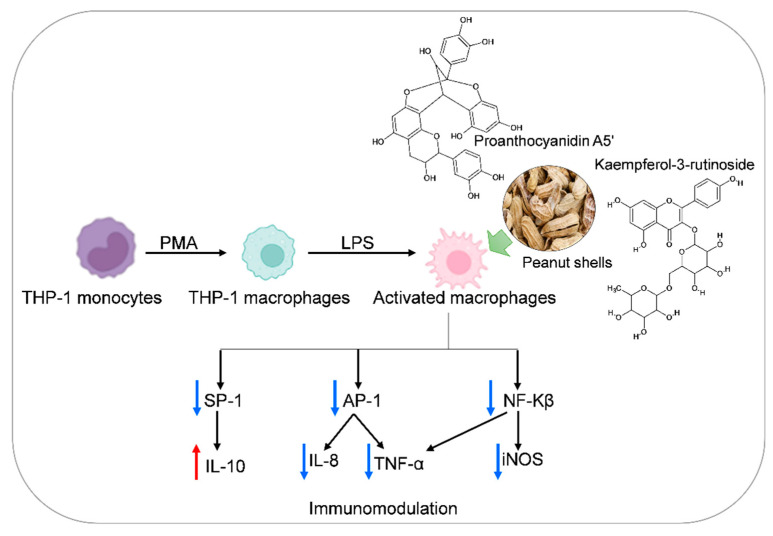
Suggested molecular mechanism of peanut shells bioactive metabolites as immunomodulatory.

Intriguingly, Kaempferol rutinoside and proanthocyanidin A5’ were found to be the most promising potential iNOS inhibitors. Kaempferol rutinoside has been reported to possess wound healing [66], accelerating keratinocyte cell migration [67], potentiated cell migration by induction of lamellipodia and filopodia formation via an increase in active Rac1-GTP [68], treating acute orofacial pain [69], in addition to anti-inflammatory effect in lipopolysaccharide-treated mouse macrophage RAW264.7 cells [58]. Improvement of vascular health by the consumption of proanthocyanidin A5 was supported by many research studies. For example, this effect is due to an increase in NO production, which leads to vasodilation, inhibition of platelet aggregation, decreased low-density lipoproteins (LDL) sensitivity to oxidization, in addition to its antioxidant, anticancer, and anti-inflammatory effect [70]. Based on the above findings, we recommend further advanced *in vitro* as well as *in vivo* evaluations of these compounds in order to obtain an effective drug member acting as a potential inhibitor of the iNOS receptor (Figure 8). This may provide us with an immunomodulator and/or anti-inflammatory drug candidate targeting the iNOS receptor effectively. Furthermore, the studied compounds may be either alone or in combinations.

## 5. Conclusions

This study contributes to an in-depth search for the nut shells immunomodulatory activity. The observed immunomodulatory and anti-inflammatory activity, as well as antioxidant capability, are attributed to their high flavonoid and phenolic contents. Peanut shells show the most significant results in suppressing the production of pro-inflammatory cytokines such as TNF-α and IL-8 in THP-1 stimulated by LPS. Furthermore, it reduces the inflammation-related enzymes (iNOS and COX-2) as well as a transcription factor (AP-1, SP-1, and NF-κB), which reveal its potential immunomodulatory effect resulting in disease prevention and normal tissue homeostasis. Peanut shell metabolic profile uniquely presented by epicatechins and flavonoids, which may contribute to the potential immunomodulatory and antioxidant activities. Kaempferol rutinoside and proanthocyanidin A5’ were found to be the most promising members as potential iNOS inhibitors with superior binding scores compared to that of the docked co-crystallized inhibitor. Therefore, we recommend further *in vitro* and *in vivo* studies on the previously mentioned candidates either alone and/or in combinations to provide an immunomodulator and/or anti-inflammatory drug candidate targeting the iNOS receptor effectively. 

## Figures and Tables

**Table 1:** The biochemical effect of different shells on IL-1β, IL-6, IL-8, IL-10 and TNF-α levels.

Groups	IL-1β	IL-6	IL-8	IL-10	TNF-α
Negative Control	50.1 ± 5.9	41.6 ± 5.2	61.9 ± 2.25	165 ± 3.67	35.4 ± 2.1
Positive Control	289 ± 11 *	347 ± 11.4 *	610 ± 75.4 *	29.3 ± 2.5 *	273 ± 7.26 *
Peanut shells	64.4 ± 4.6 ^#^	113.2 ± 5.46 *^#^	135.6 ± 13.2 *^#^	121 ± 11.2 ^#^	83.4 ± 4.35 *^#^
Almond shells	171.1 ± 6.2 *^#@^	213.7 ± 21.4 *^#@^	458 ± 32 *^#@^	50.6 ± 1.97 *^@^	166 ± 13.9 *^#@^
Hazelnut shells	166.6 ± 5.6 *^#@^	212 ± 19.2 *^#@^	429 ± 41.5 *^#@^	52.9 ± 2.25 *^@^	178 ± 10.5 *^#@^
Walnut shells	204.2 ± 7.21 *^#@^	242 ± 28.5 *^#@^	505 ± 19.8 *^#@^	50.7 ± 1.91 *^@^	149 ± 1.93 *^#@^

Results were expressed as mean ± SD and analysis was performed using one-way ANOVA followed by Tukey post hoc test. * Significant from negative control at *p* < 0.0001, ^#^ Significant from Positive control at *p* < 0.0001, ^@^ Significant from Peanut shells at *p* < 0.001.

**Table 2:** Binding scores and interactions of the selected compounds accumulating in peanut shells towards the binding pocket of iNOS compared to the co-crystallized inhibitor, CLW.

Compound	S ^a^	RMSD ^b^	Amino Acid Bond	L ^c^
Epicatechin	−5.66	0.69	Met368/H-acceptor	3.16
**Epicatechin methylgallate**	**−7.54**	**1.92**	**Trp366/H-pi** **Met368/H-donor**	**3.76** **4.25**
**Proanthocyanidin A1**	**−7.73**	**1.90**	**Met368/H-acceptor**	**3.01**
**Proanthocyanidin A5’**	**−8.67**	**0.72**	**Trp366/H-donor** **Glu371/H-donor**	**2.85** **3.02**
**Kaempferol glucoside**	**−7.82**	**1.31**	**Trp366/H-acceptor** **Trp366/H-donor** **Trp366/H-donor**	**3.10** **3.13** **3.24**
**Kaempferol rutinoside**	**−9.96**	**1.38**	**Trp366/H-donor** **Met349/H-donor** **Met349/H-donor**	**2.82** **3.36** **3.57**
**Quercetin galactoside**	**−7.47**	**0.71**	**Trp366/H-donor**	**2.73**
Hydroxy-methoxyflavone	−5.87	1.34	Met368/H-acceptorPro344/pi-H	3.343.74
Tetrahydroxy prenylflavone	−7.02	1.06	Trp366/H-donorCys194/pi-H	2.983.89
Trihydroxy methoxyprenylisoflavone	−7.04	1.04	Trp366/pi-H	4.23
6-C-Prenylapigenin	−6.70	1.06	Met368/H-acceptorTrp366/pi-H	3.054.28
Tryptophan	−5.51	1.44	Trp366/H-donor	2.81
Proline	−4.60	0.72	Trp366/H-acceptorMet428/H-donor	3.544.49
**CLW**	**−4.78**	**1.46**	**Trp366/H-donor** **Met368/H-acceptor**	**2.97** **3.04**

**^a^ S**: Score of a compound inside the protein binding pocket (Kcal/mol), **^b^ RMSD**: Root Mean Squared Deviation between the predicted pose and the crystal structure. ^**c**^**L**: Length of the bond (Å).

**Table 3:** Three-dimensional binding interactions and positioning between the most promising compounds accumulating in peanut shells at the iNOS-binding pocket compared to CLW (docked).

Compound	3D Interactions	3D Positioning
**CLW**	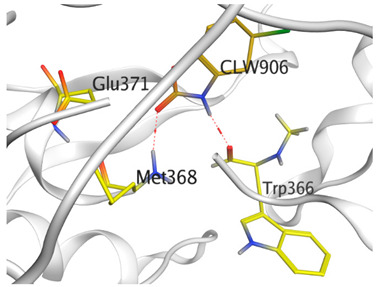	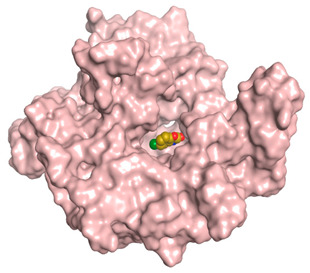
**Epicatechin-methyl gallate**	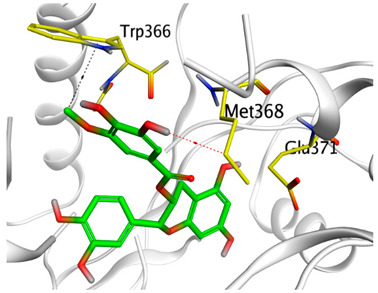	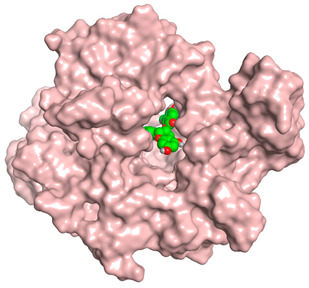
**Proanthocyanidin A1**	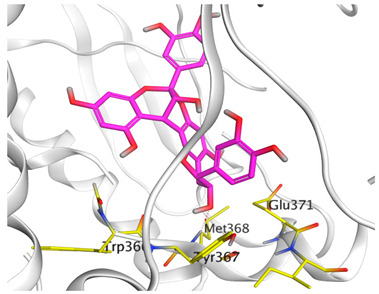	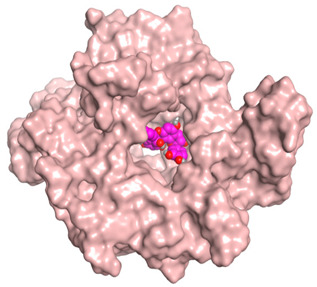
**Proanthocyanidin A5’**	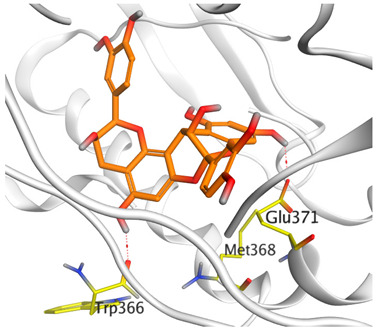	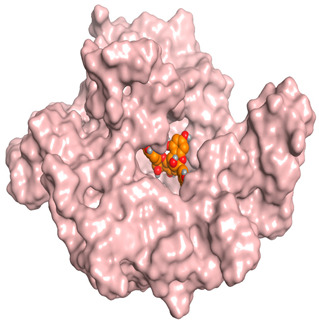
**Kaempferol glucoside**	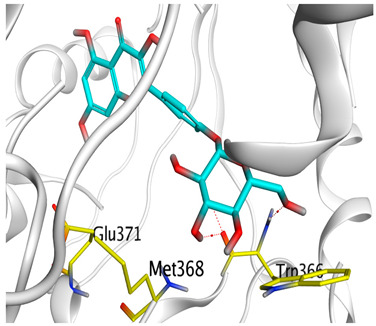	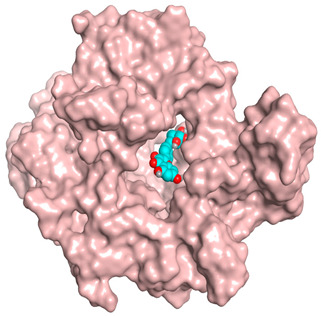
**Kaempferol rutinoside**	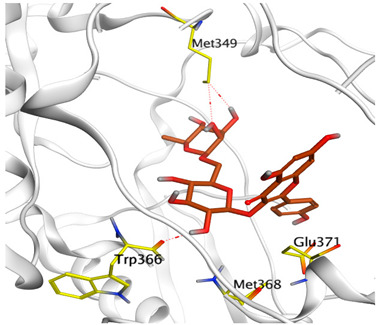	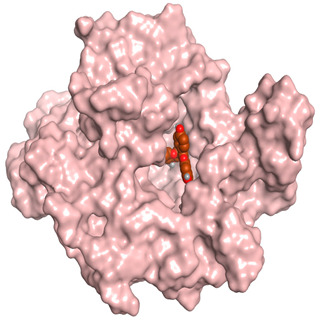
**Quercetin galactoside**	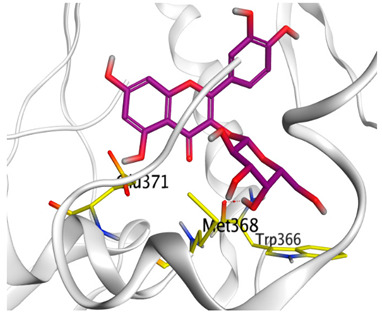	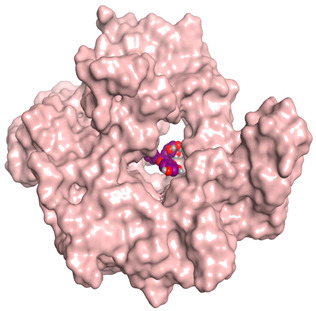

The red dash represents H-bonds and the **black** dash represents H-pi interactions.

## Data Availability

The data used to support the findings of this research were included in the manuscript and the supplementary material.

## Supplementary Materials

The following supporting information can be downloaded at: https://www.mdpi.com/article/10.3390/antiox11030462/s1, Table S1: The sequence of primer used in the study; Table S2: Metabolites identified from nut shell extracts based on the UPLC/MS data; Table S3: 2D pictures describing the binding interactions of the selected compounds accumulating in peanut shells (1–13) towards the binding pocket of iNOS compared to the co-crystallized inhibitor, CLW (14), Figure S1: Correlation between the total phenolics contents and antioxidant potential of nut shell extracts; Figure S2: Effect of the nut shell extracts on the THP-1 cell viability using the MTT assay.
