# Supplementary material for: Potential Valorization of Edible Nuts By-Products: Exploring the Immune-Modulatory and Antioxidants Effects of Selected Nut Shells Extracts in Relation to Their Metabolic Profiles
Source: Antioxidants (Basel). 2022 Feb 25;11(3):462. doi: 10.3390/antiox11030462 (PMC8944461; doi:10.3390/antiox11030462)
Supplement: Supplementary file 1 [file antioxidants-11-00462-s001.zip › antioxidants-1613470-supplementary.pdf]

Table S1. The sequence of primer used in the study

Target gene (HGNC ID)	Primer name	Primer sequence from (5' to 3')
NF-Kβ (7794)	NF-KB F<br>NF-KB R	5'- AGGCAAGGAATAATGCTGTCCTG -3'<br>5'- ATCATTCTCTAGTGTCTGGTTGG -3'
COX-2 (9605)	COX-2 F<br>COX-2 R	5’-ATCATTCACCAGGCAAATTGC-3’<br>5’- GGCTTCAGCATAAAGCGTTTG -3’
iNOS (7873)	iNOS F<br>iNOS R	5’- GCTCTACACCTCCAATGTGACC -3’<br>5’- CTGCCGAGATTTGAGCCTCATG -3’
SP-1 (11205)	SP-1 F<br>SP-1 R	5’- TTGAAAAAGGAGTTGGTGGC -3’<br>5’- TGCTGGTTCTGTAAGTTGGG -3’
AP-1 (1258)	AP-1 F<br>AP-1 R	5’- TGTTACTCTTTCCCTCTCTGCTGG -3’<br>5’- GCCACATACAACCAGGGAGTCA -3’
	β-actin F<br>β-actin R	5’-GTGACATCCACACCCAGAGG-3’<br>5’-ACAGGATGTCAAAACTGCCC-3’

Table S2. Metabolites identified from nut shell extracts based on the UPLC/MS data.

Number	Putative compound Name	Formula	RT (min.)	Detected m/z	Delta (ppm)
1.	3-carboxy-2-hydroxy Phenylalanine	C10H11NO5	3.28	226.0694	-11.8
2.	4-Pyridoxic Acid	C8H9NO4	4.78	184.061	-2.78
3.	6-C-Prenylapigenin	C20H18O5	13.3	383.1138	0.53
4.	6-Hydroxynicotinic Acid	C6H5NO3	2.68	140.0343	-6.92
5.	alpha-Ketoglutaric acid	C5H6O5	1.07	191.0192	-2.86
6.	Apigenin	C15H10O5	7.88	271.0615	0.98
7.	Benzyl gentiobioside	C19H28O11	4.81	477.1617	0.73
8.	Butanediol apiosylglucoside	C15H28O11	3.04	429.1616	0.46
9.	Butanol Apiofuranosyl glucopyranoside	C15H28O10	4.03	413.1669	1.09
10.	Caftaric acid	C13H12O9	7.83	358.0571	8.12
11.	Catechin	C15H14O6	4.98	335.0756	-4.92
12.	Chicoric acid	C22H18O12	6.64	475.0848	-7.2
13.	Citric acid	C6H8O7	1.12	193.0348	-3.19
14.	Coumaric acid	C9H8O3	4.86	165.0549	-5.13
15.	Coutaric acid	C13H12O8	4.83	297.0607	-3.1
16.	Cyanidin 3-(6-acetylgalactoside)	C23H22O12	6.43	491.12	1.01
17.	Dicaffeoyl quinolactone	C25H22O11	7.92	544.1335	20.78
18.	Dihydroxy dimethoxy prenylflavanone	C22H24O6	4.87	385.1635	-5.5
19.	Dihydroxy prenylflavanone	C20H20O5	12.97	385.1298	1.34
20.	Dihydroxy-1-benzopyran-2-one	C9H6O4	4.97	179.0343	-3.63
21.	Dihydroxy-methoxy-hydroxybenzyldihydrochalcone	C23H22O5	15.31	379.1476	-19.82
22.	Epicatechin isomer	C15H14O6	7.87	291.0876	0.49
23.	Epicatechin methylgallate	C23H20O10	6.21	501.102	-3.77
24.	Epicatechin-catechin	C30H24O12	8.28	577.1477	21.76
25.	Epigallocatechin methylgallate	C23H20O11	3.76	473.1138	10.28
26.	Eriodictyol dimethyl ether	C17H16O6	12.84	317.1033	0.76
27.	Eriodictyol glucoside	C21H22O11	5.71	451.1185	-13.56
28.	Fertaric acid	C14H14O9	5.23	327.0726	1.46
29.	Feruloylglucose trihydroxy methylbutylglycoside	C21H30O12	6.22	475.1923	21.51
30.	Galactopinitol B	C13H24O11	0.67	401.1304	0.92
31.	Galloylglucose	C13H16O10	2.98	333.081	-5.27
32.	Glutamic Acid	C5H9NO4	0.66	148.0607	-5.62
33.	Glutathione (reduced)	C10H17N3O6S	1.14	308.099	22.04
34.	Gravelliferone	C19H22O3	13.78	299.159	-20.96
35.	Hesperidin	C28H34O15	7.33	611.1995	2.16
36.	Hydroxy jasmonic acid glucoside	C18H28O9	5.64	389.182	0.85
37.	Hydroxy tetramethoxystilbene	C17H18O5	7.87	347.114	1.03
38.	Hydroxybdihydrojasmonic acid glucoside	C18H30O9	5.94	391.1977	0.94
39.	Hydroxy-dimethoxyflavanone rhamnoside	C23H26O9	1.06	491.1634	15.28
40.	Hydroxykaempferol	C15H10O7	9.4	303.0511	0.3
41.	Hydroxylinolenic acid	C18H32O3	14.22	297.2437	0.45
42.	Hydroxy-methoxyflavone	C16H12O4	11.8	269.0821	0.71
43.	Isocitric acid	C6H8O7	1.28	193.0348	-3.19
44.	isopropylapiosyl glucoside	C14H26O10	3.3	399.151	0.58
45.	Kaempferol glucoside	C21H20O11	7.41	449.1094	0.98
46.	Kaempferol rutinoside	C27H30O15	5.67	595.1571	-16.36
47.	Kushenol C	C25H26O7	5.79	439.1739	-5.25
48.	Linalool oxide primeveroside	C21H36O11	6.1	509.2242	0.43
49.	Linalool xylosyl-glucoside	C21H36O10	9.35	493.2297	1.33
50.	Linolenic acid	C18H30O2	14.57	279.2331	0.5

51.	luteolin	C15H10O6	9.42	287.0562	0.36
52.	Luteolin diglucoside	C15H10O6	9.4	287.0562	0.25
53.	Malvidin malonylglucoside	C26H26O15	6.47	579.1362	1.19
54.	Methoxy Kaempferol	C16H12O7	8.98	317.0666	-0.18
55.	Methyl epicatechin glucuronide isomer	C22H24O12	6.52	481.1334	-3.72
56.	Methylkaempferol	C16H12O6	10.8	301.072	0.66
57.	Moracin I	C20H20O4	12.84	369.1343	-0.06
58.	Myricetin trimethyl ether	C18H16O8	7.43	361.0929	0.01
59.	N-Acetyl-Proline	C7H11NO3	3.72	202.0715	-3.09
60.	Naringenin neohesperidoside	C27H32O14	7.3	581.1879	0.6
61.	Octadecadienoic acid	C18H32O2	13.9	325.2383	-0.37
62.	Octadecendioic acid	C18H34O4	13.43	315.2542	0.41
63.	Oleic acid	C18H34O2	14.79	327.2543	0.77
64.	Parvisoflavone A	C20H16O6	12.62	353.1032	0.51
65.	Phenylalanine	C9H11NO2	3.32	210.0768	-1.68
66.	PI (18:2/0:0)	C27H49O12P	13.89	597.3131	14.35
67.	Proanthocyanidin A1	C30H24O12	9.16	577.1257	-16.42
68.	Proanthocyanidin A5'	C30H24O12	6	622.148	24.44
69.	Proline	C5H9NO2	0.74	116.0706	-9.41
70.	Pumilaisoflavone	C27H28O7	5.78	509.1804	-2.64
71.	Quercetin acetyl-glucoside	C23H22O13	7.34	507.1141	-0.55
72.	Quercetin galactoside	C20H18O12	7.37	495.0756	-4.86
73.	Quercetin galacturonide	C21H18O13	5.9	479.0856	5.26
74.	Quercetin glucoside	C21H20O12	7.01	465.1045	1.45
75.	Quercetin methyl ether glucoside	C22H22O12	6.77	479.1202	1.48
76.	Quercetin-rhamnose-glucoside	C27H30O16	6.79	611.1583	-5.62
77.	Resveratrol glucoside	C20H22O8	6.16	391.1404	1.46
78.	S-Adenosylhomocysteine	C14H20N6O5S	2.49	429.1252	12.57
79.	Secoisolariciresinol glucoside	C26H36O11	7.27	569.2247	1.24
80.	Tetrahydroxy prenylflavone	C20H18O6	12.44	355.1189	0.61
81.	Tetrahydroxyisoflavanone	C15H12O6	9.17	333.0617	0.35
82.	Tetrahydroxy-prenylflavone	C20H18O6	13.05	355.119	0.86
83.	Tetramethoxychalcone glucoside isomer	C25H30O11	1.09	551.1732	-7
84.	Theviridoside	C17H24O11	4.9	405.1404	0.51
85.	Trehalose	C12H22O11	0.71	387.1146	0.39
86.	Trihydroxy methoxydihydrochalcone	C16H16O5	7.25	333.0985	1.61
87.	Trihydroxy methoxyprenylisoflavone	C21H20O6	13.16	369.1343	-0.06
88.	Trihydroxydihydro-linalyl oxide glucopyranoside	C16H30O10	16.02	383.1895	-7.11
89.	Trihydroxyflavanone	C15H12O5	10.35	317.067	1.07
90.	Trihydroxy-octadecenoic acid	C18H34O5	11.41	331.2494	1.09
91.	Tryptophan	C11H12N2O2	4.39	249.0853	-11.11
92.	Valine	C5H11NO2	1	162.0766	-3.78
93.	Vanillin acetate	C10H10O4	5.28	195.0657	-2.81

Table S3. 2D pictures describing the binding interactions of the selected compounds accumulating in peanut shells (**1-13**) towards the binding pocket of iNOS compared to the co-crystallized inhibitor, CLW (**14**).

No.	Compound	2D binding interactions
**1**	Epicatechin	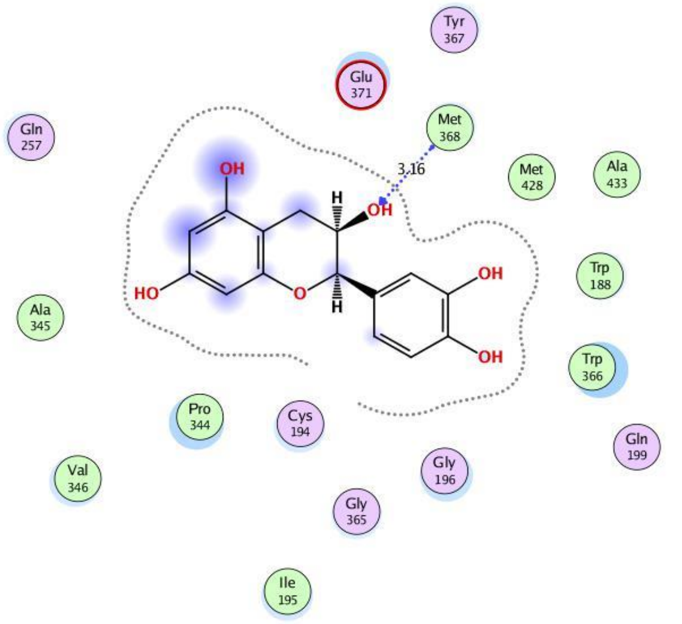
**2**	Epicatechin methyl gallate	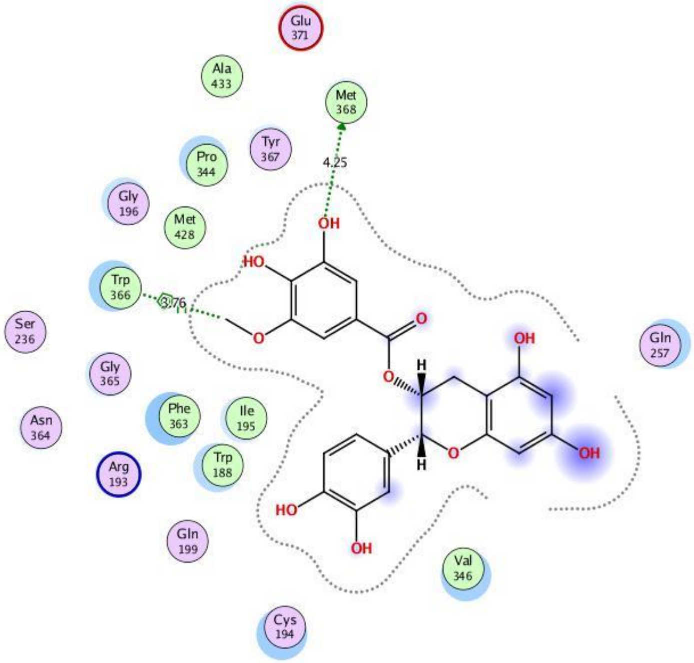

<table>
<tr><td>3</td><td>Proanthocyanidin A1</td><td>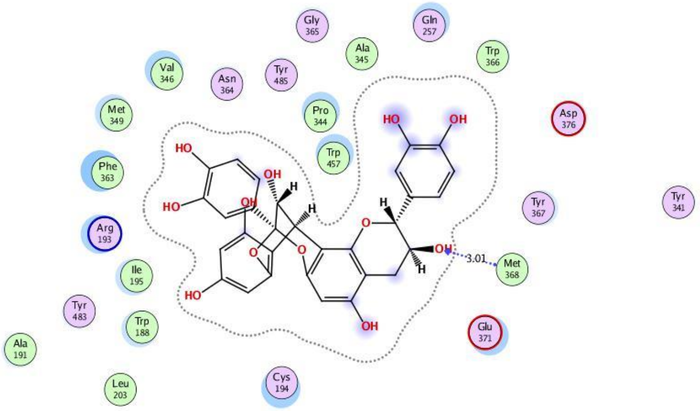
</td></tr>
<tr><td>4</td><td>Proanthocyanidin A5'</td><td>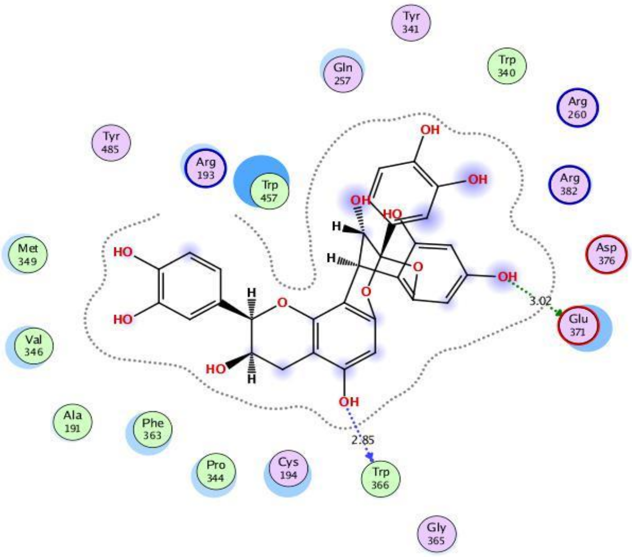
</td></tr>
<tr><td>5</td><td>Kaempferol glucoside</td><td>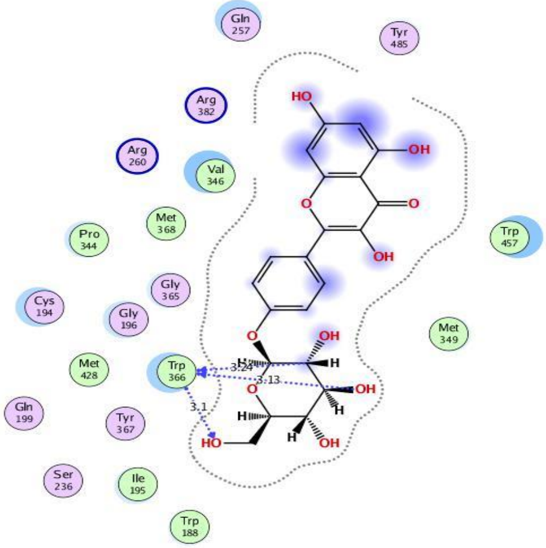
</td></tr>
</table>

**6**	Kaempferol rutinoside	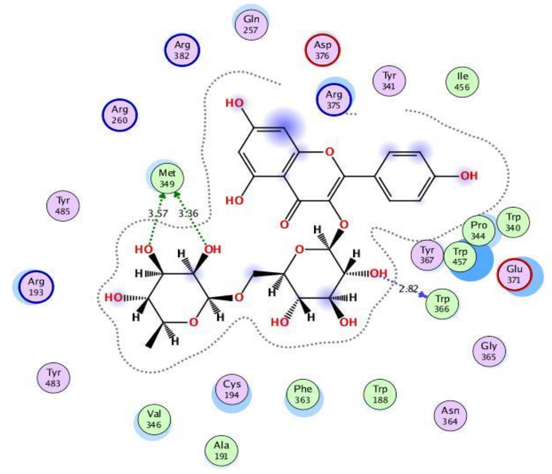
**7**	Quercetin galactoside	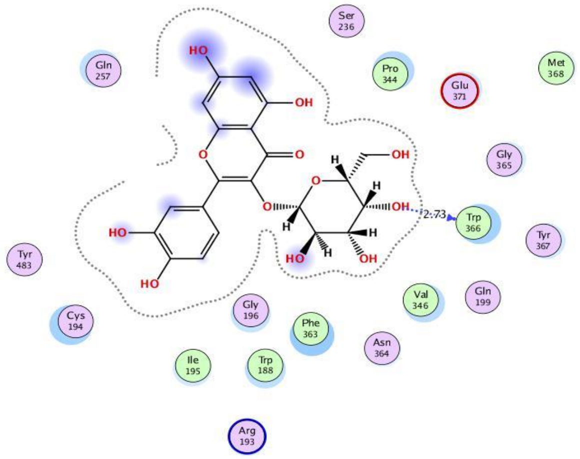
**8**	Hydroxy-methoxy flavone	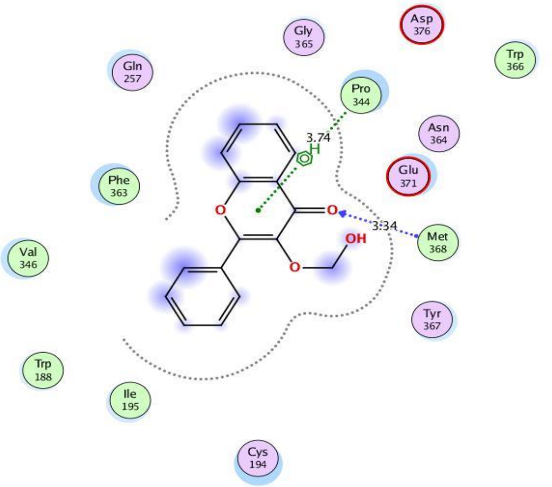

**9**	Tetrahydroxy prenylflavone	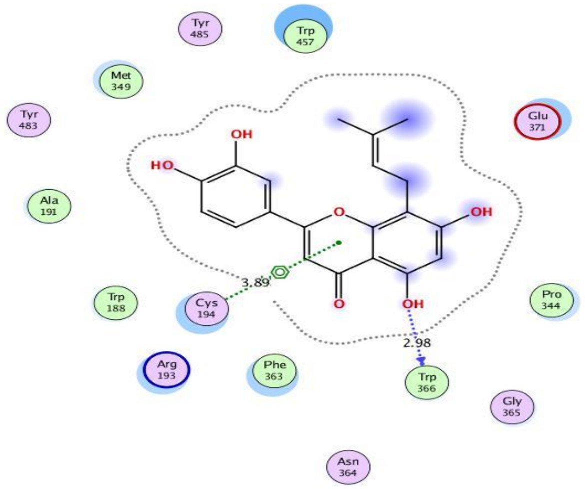
**10**	Trihydroxy methoxy-prenyl isoflavone	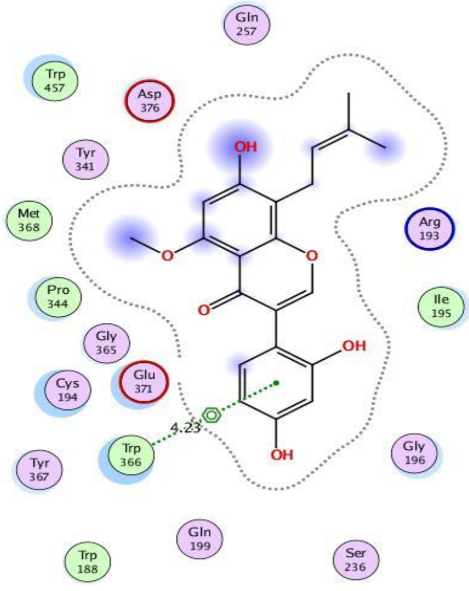
**11**	6-C-Prenylapigenin	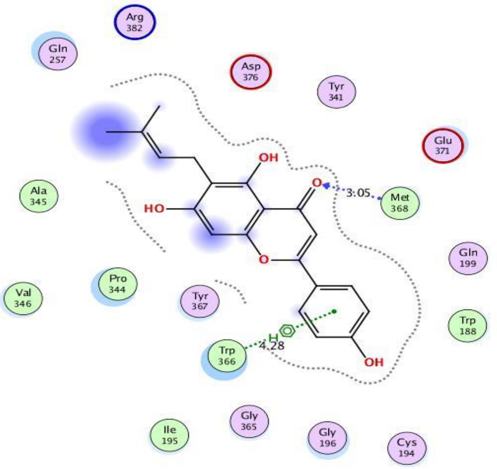

<table>
<tr><td>12</td><td>Tryptophan</td><td>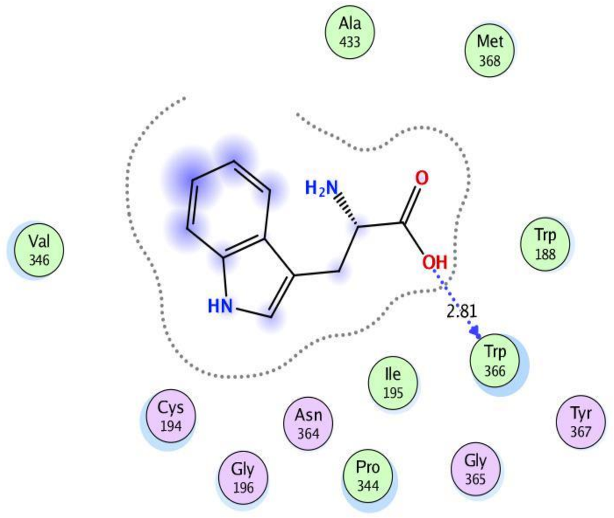
</td></tr>
<tr><td>13</td><td>Proline</td><td>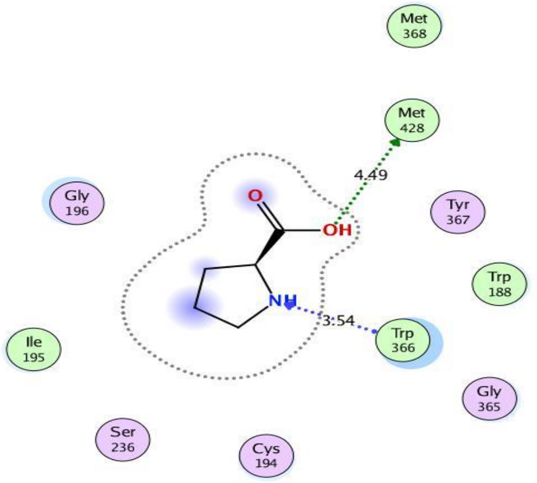
</td></tr>
<tr><td>14</td><td>CLW
(docked)</td><td>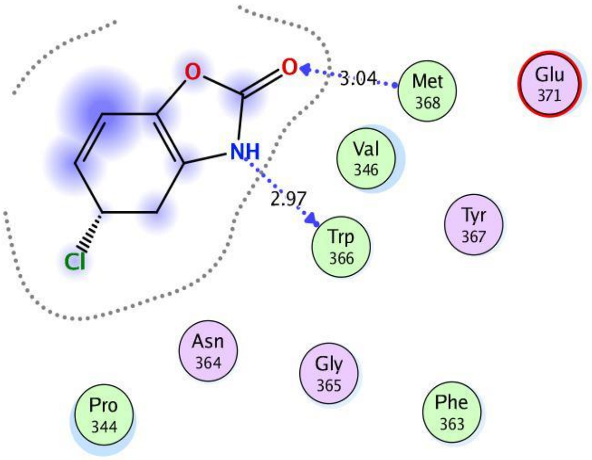
</td></tr>
</table>

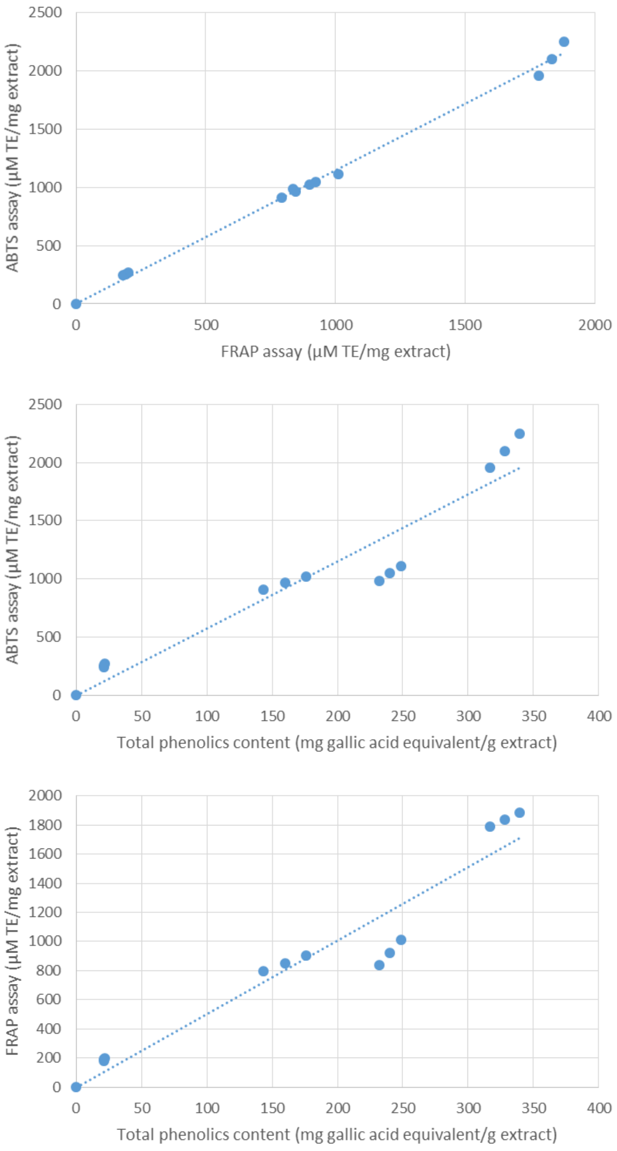

Figure S1. Correlation between the total phenolics contents and antioxidant potential of nut shell extracts

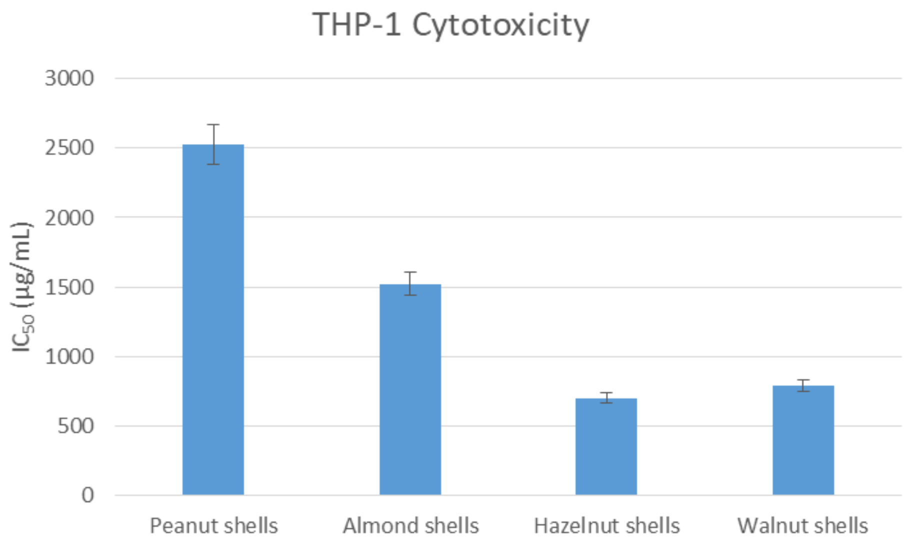

Figure S2. Effect of the nut shell extracts on the THP-1 cell viability using the MTT assay.
